# Acceptability of Online Self-Help to People With Depression: Users’ Views of MoodGYM Versus Informational Websites

**DOI:** 10.2196/jmir.2871

**Published:** 2014-03-28

**Authors:** Justine Schneider, Pooria Sarrami Foroushani, Paul Grime, Graham Thornicroft

**Affiliations:** ^1^Institute of Mental Health & School of Sociology & Social PolicyUniversity of NottinghamNottinghamUnited Kingdom; ^2^Australian Institute of Health InnovationUniversity of New South WalesSydneyAustralia; ^3^Cambridge Health at WorkCambridge University HospitalsUniversity of CambridgeCambridgeUnited Kingdom; ^4^David Goldberg CentreHealth Service and Population ResearchInstitute of Psychiatry, King's CollegeLondonUnited Kingdom

**Keywords:** computers, cognitive therapy, method acceptability, depression, workplace, qualitative evaluation

## Abstract

**Background:**

Little is known about the factors that influence acceptability of and adherence to online psychological interventions. Evidence is needed to guide further development of promising programs.

**Objective:**

Our goal was to investigate users’ views of two online approaches to self-help for depression: computerized cognitive behavior therapy (cCBT) and informational websites, in a workplace context. Computerized CBT offers an inexpensive and accessible alternative to face-to-face therapy, and employers have an interest in reducing the working time lost to depression or stress. Yet little is known about how employees, who have actual experience of using online approaches, judge the intervention as a process.

**Methods:**

The qualitative data reported here were collected within an online randomized controlled trial whose participants had diagnosable depression. The experimental intervention was a 5-week cCBT program called MoodGYM, and the control condition was five informational websites about mental health. Data were collected via online questionnaires. There was no evidence of the superiority of either in terms of treatment outcomes. In parallel, using brief rating scales and open-ended questions designed for this purpose, we examined the relative acceptability of each approach over time, including perceptions of cCBT compared to seeing a health care professional.

**Results:**

At least 60% of participants held online therapy to be at least as acceptable as seeing a professional about mental health issues, and they were more likely to retain this opinion over time if they used the interactive program, MoodGYM, rather than informational websites alone. Barriers to cCBT use fell into four categories: intrinsic, intrapersonal problems; extrinsic technical problems; generic issues mostly pertaining to perceptions of cCBT; and specific issues about the intervention or control condition. These indicate strategies for improving engagement.

**Conclusions:**

As first-aid for mild to moderate mental health problems, evidence-based computerized approaches have broad acceptability. This could be increased by attending to the barriers noted here and by proactively managing users’ expectations at individual and organizational levels. The findings have implications for occupational health providers and others addressing the needs of working-age adults with depression. They also raise methodological issues for online research.

**Trial Registration:**

International Standard Randomized Controlled Trial Number (ISRCTN): 24529487; http://www.controlled-trials.com/ISRCTN24529487 (Archived by Webcite at http://www.webcitation.org/6O8cCL4mh).

## Introduction

The demand for cognitive behavioral therapy (CBT) is such that the supply of therapists for face-to-face counseling is insufficient. Thus, the attraction of computerized approaches lies in low cost, ease of access, broad acceptability [1], and the possibility that online provision is relatively free of the stigma attached to formal mental health services and their users [2]. The evidence for effectiveness is growing rapidly in relation to a handful of interventions designed to address common mental disorders [3-6]. This demand is driven partly by employers’ awareness of the impact on productivity of depression in the workplace [7].

Maxwell proposed six indicators of service quality: acceptability, efficacy, safety, equity, efficiency, and accessibility. Acceptability was defined as responses to the following questions: What do service users think of a service? How would they feel if the service was the most costly? Are there any issues of privacy or confidentiality? [8] As Kaltenthaler et al assert, the acceptability of an intervention is crucial to appraising its effect. If trial participants are reluctant to engage fully with an intervention, or if they drop out in disproportionate numbers, the internal or external validity of the results may be compromised [4]. Acceptability is also vital to the implementation of evidence-based treatment in practice, since low take-up will impair efficacy. People with depression may face particular difficulty in complying with treatment due to their low mood [9]. Contextual factors, subjective beliefs, and technical problems may also affect engagement with an intervention [10]. Therefore, in evaluating novel approaches, explicit questions need to be asked about user expectations, experience, and satisfaction: this is what is meant by acceptability. While it is crucial to understand the underlying reasons for (un)acceptability of computerized treatments of depression, there is still limited information in this regard.

The aim of this paper is to examine the acceptability of one form of computerized cognitive behavioral therapy (cCBT)—online self-help for depression—in the context of a workplace trial where the data were largely collected online. It reports on a study that explored two different dimensions: acceptability (data presented here), and effectiveness of computerized treatments (data presented by Phillips et al, 2013) [11]. One problem with online data collection is retaining subjects in the trial; the present study is no exception, with a relatively high dropout rate (45% at 6 weeks). However, those people who completed are a group whose opinions may suggest improvements, both to the interventions tested and to the method of data collection.

MoodGYM was chosen for this study because it is freely available on the Internet [12]. Its website describes MoodGYM as “A free self-help program to teach cognitive behavior therapy skills to people vulnerable to depression and anxiety”. There is evidence of effectiveness from a community-based trial, in which users were supported by weekly phone calls [13,14]. In that study, 79% completed the intervention. Although the dropout was greater from MoodGYM than from the control group, the effect of MoodGYM endured longer than the other intervention, self-help. One recent UK study indicates that MoodGYM is effective in promoting general mental well-being in a non-clinical population [15].

This paper addresses the participants’ views about the process of online interventions, in order to guide the design of future developments and their evaluation. The present study indicated that both the participants who received the interactive online intervention and those who received passive information provision improved equally over time. Although the interactive online self-help did not display superior effectiveness, the two approaches may still differ in terms of acceptability. In any case, it is important to explore acceptability of computer methods in comparison with face-to-face interventions. Considering the large numbers of people affected by common mental health problems, online self-help, in either interactive or passive forms, may be one means to reduce the demand for face-to-face therapy and cut costs.

## Methods

### Overview

A workplace setting was chosen because of the prevalence and impact on productivity of mild to moderate depression [16] and early evidence of the benefits of cCBT [4,5,13]. A phase III, two-arm, parallel randomized controlled trial (RCT) whose main outcome was total score on the Work and Social Adjustment Scale (WSAS) was implemented using an online questionnaire. A list was produced by the Nottingham Clinical Trials Unit to allow simple (unrestricted) randomization. Statistical analysis shows improvement in both arms but no difference between the experimental and attentional control groups [11]. Acceptability of the intervention was investigated qualitatively by incorporating five Likert-style questions, an open-ended question about likes and dislikes, and four comparative questions designed for the study and described below.

Ethical approval was granted by Australia National University ethics committee and a favorable opinion was given by the Derby local research ethics committee.

### Recruitment

The study was promoted in three large UK employers: two private enterprises (telecommunications and transport) and one public sector employer (health) between September 2009 and May 2011. The first employer to join the trial actively promoted the opportunity to staff through internal communications and ultimately recruited 396 participants. The second provided it mainly but not exclusively through their occupational health department personnel, who identified people likely to qualify for it and recruited 100. The third employer had a hands-off approach, simply publicizing it on an intranet, and recruited 141 participants. The numbers do not reflect the effectiveness of the different approaches to recruitment because the workforces also differed in size. Assurances were given that participation was voluntary and that the study was independent of the employers, so that respondents’ identities and data would be confidential.

### Inclusion Criteria

Online screening of potential participants offered the option of joining the trial if they were over age 18 and met the following criterion of likely depression: on Patient Health Questionnaire-9 (PHQ-9) [17], the employee scored 2 or more on 5 of the 9 items, including 2 or more on item 1 (little interest in doing things) or item 2 (feeling hopeless). To be eligible, the employee also had to confirm that at least one of the items identified as a problem for them made it difficult to work, take care of things at home, or get along with other people. People who did not meet the PHQ threshold, or whose functioning at work or home was not impaired, or who were unwilling to give consent and be contacted by telephone were excluded from the trial.

### Procedures

Participants in both the MoodGYM and the control groups received six weekly telephone calls from clinical studies officers (CSOs) of the UK Mental Health Research Network, to screen for risk of self-harm, deal with technical problems, and gather data about service use (not presented here). Participants input the rest of their own data through a research portal, which in effect screened them for eligibility, took consent, delivered the interventions, and administered baseline and follow-up measures. Participants were kept “blind” to their status as intervention or control group members, by referring to the trial’s focus as “self-help for stress”, which describes both conditions.

**Figure 1:**
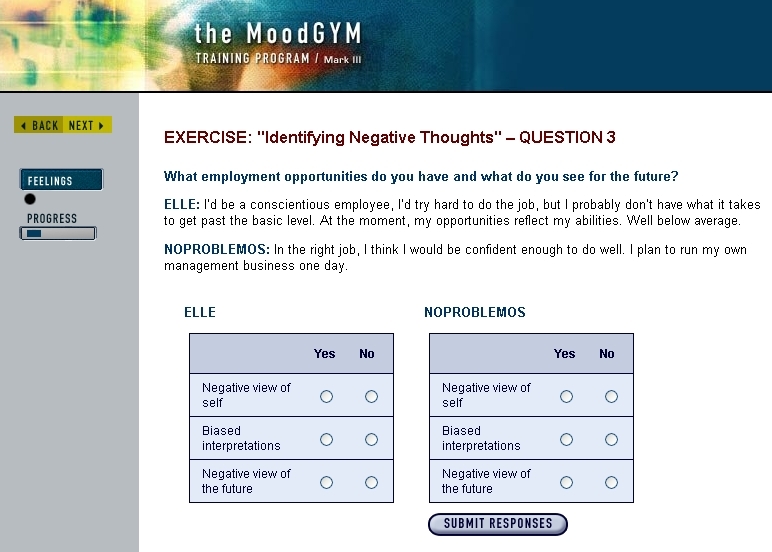
Example page from experimental website.

**Figure 2:**
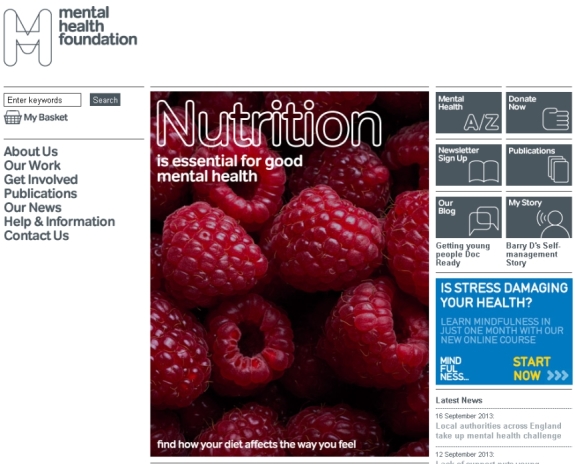
Example page from one control-arm website.

The MoodGYM intervention is a modularized course developed at Australia National University. It is designed to last 5 weeks with assessments in the sixth week, although participants proceed at their own pace. The websites selected for the “attentional control” group were judged to be reliable sources of information about mental health problems. They were known to the chief investigator from a previous review of self-help in mental health, had been identified for teaching purposes as suitable materials to inform UK health and social care professionals, and included the British Broadcasting Corporation (the BBC), National Health Service (NHS) Direct, and Royal College of Psychiatrists information pages. A different website was recommended each week by automated emails. Sample screenshots of the intervention and control group are presented in Figures 1 and 2.

### Measures of Acceptability

**Figure 3:**
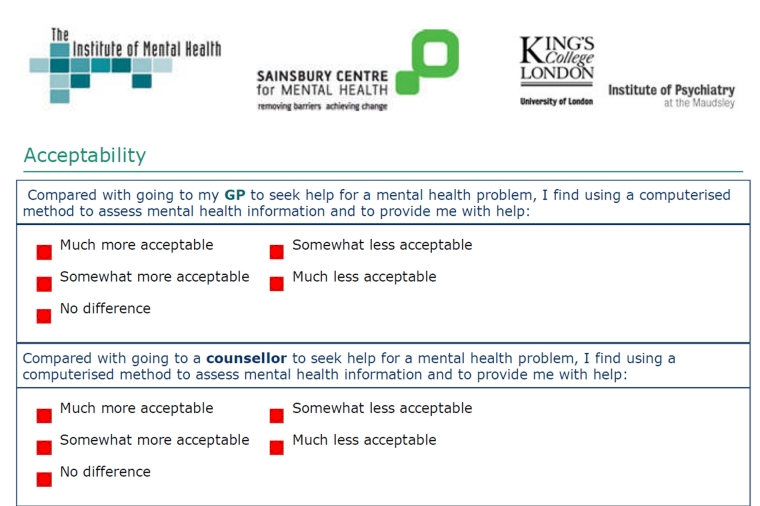
Questionnaire screenshot.

“Acceptability” is used here to convey the users’ appraisal of online self-help. Lacking much prior work in this area and bearing in mind the self-selected nature of the sample, we explored three perspectives on what we broadly call acceptability using questions devised by the study team. These three tools were piloted along with the rest of our instruments. First, participants were asked in the online questionnaire to judge the importance of five statements that might be taken to reflect aspects of acceptability (not important, important, very important). They were also given an open-ended statement that invited further comments on reasons to like or dislike help via the Internet. Finally, we sought to investigate the relative acceptability of online self-help by asking participants to compare it with their perceptions of personal consultations with a range of health care professionals: general practitioner (GP), counselor, psychologist, and psychiatrist (see Figure 3). We did not ask whether respondents had prior experience of such services for their mental health.

In our analysis, in order to differentiate between anticipated or imagined preferences and actual impressions of the online approaches, baseline results were taken to reflect users’ *expectations* of the process, as compared to 6- and 12-week results, when people reported their actual *experiences* of using the online resources.

### Quantitative Analysis

Agreement with structured statements about acceptability and comparisons with professional help were analyzed using descriptive statistics. Change in individual ratings over time was explored using paired *t* tests. *T* tests of independent means were used order to investigate (1) differences between intervention and control groups and (2) differences between those people who started out with positive or negative expectations of the online approach. Probability of completing the structured questions about acceptability at 6 and 12 weeks was investigated by study arm using chi-square tests.

### Qualitative Analysis

Responses to open-ended questions were imported into MS Excel, sorted, and coded using a grounded theory approach [18]. The categories were not pre-set but evolved from the data, which were iteratively reviewed and reclassified to reduce the findings to concise, meaningful, and mutually exclusive topics. Once categories emerged in this way, the respondents’ comments were allocated and counted. In some categories, they were also coded as broadly negative, positive, or neither.

## Results

### Summary

There were 9305 visits to the website: 1715 went on to give informed consent and complete the screening for eligibility, 1111 were eligible because of their level of depression, and 637 complied with the requirements to proceed, which included giving a contact telephone number and completing the baseline questionnaire. Of these participants in the trial, 56% (359/637) responded to the structured questions 6 weeks later and 36% (231/637) responded to them at 12 weeks. See Multimedia Appendix 1 for a flow diagram.

### Sample Profile

The study sample is described in Table 1. There were no major differences; subjects had similar mean age, years in school, occupations, and levels of alcohol consumption compared across both arms. More males were randomized to control than MoodGYM (50.2%, 160/319 vs 42.8%, 136/318), and more females were randomized to MoodGYM than control (55.3%, 176/318 vs 47.6%, 152/319). More single people were randomized to control than MoodGYM (25.7%, 82/319 vs 21.1%, 67/318), and more of the married/cohabiting group were randomized to MoodGYM than control (68.5%, 218/318 to 61.4%, 196/319). Comparison with workforce profiles of the employing organizations confirmed that the opportunistic approach to recruitment to the trial resulted in a sample that was not representative of the wider workforces. The major discrepancy is that people who do not work in offices are underrepresented.

**Table 1:** Characteristics of subjects according to trial allocation.

Characteristics		Control (n=319)			MoodGYM (n=318)		
		n	%		n	%	
**Gender**							
	Male	160		50.2%	136		42.8%
	Female	152		47.6%	176		55.3%
	Missing	7		2.2%	6		1.9%
Age in years, mean, SD		42.7		9.6	42.2		9.6
**Marital status**							
	Single	82		25.7%	67		21.1%
	Married/cohabiting	196		61.4%	218		68.5%
	Widowed/separated	34		10.6%	27		8.5%
	Missing	7		2.2%	6		1.9%
**Employer**							
	Telecommunications	195		61.1%	198		62.3%
	Transport	51		15.9%	49		15.4%
	Health	73		22.9%	71		22.3%
**Occupation**							
	Manager or senior official	92		28.8%	91		28.6%
	Professional	66		20.7%	63		19.8%
	Assoc. prof. or technical	33		10.3%	32		10.1%
	Admin. & secretarial	34		10.6%	52		16.3%
	Skilled trade	14		4.4%	5		1.6%
	Personal services	0		0.0%	2		0.6%
	Sales and customer service	60		18.8%	61		19.2%
	Plant & machine operative	1		0.3%	1		0.3%
	Other	19		5.9%	10		3.1%
	Missing	0		0.0%	1		0.3%
Education post-16 yrs, median, range		3		0-39	3		0-39
Interquartile range				5			5
Alcohol consumption units, median, range		5		0-140	4		0-70
Interquartile range				14			11

### Acceptability

The levels of agreement that aspects of online self-help are “important” or “very important” are summarized in Table 2. There was strong agreement with all of these assertions at baseline. At 6 weeks this remained true, but there was a significant reduction in the number of people who thought that accessing help “at any time” was an advantage *t*_359_=3.396, *P=*.001). At 12 weeks, compared to baseline, when the intervention had been completed at least one month earlier, there was a statistically significant drop in the importance given to all but one statement (“I can use the computer at my own pace”), yet a majority of respondents still regarded all five features as important. Responses to these questions were highly intercorrelated.

**Table 2:** Percentage of participants rating features “important” or “very important”.

Statement	Baseline n=654	6 weeks n=360	12 weeks n=219
I can use the computer at my own pace.	89.9%	90.8%	87.7%
Using a computer is anonymous, I don’t need to tell people about my problems.	74.8%	73.6%	73.1%
It is convenient for me to access help via the Internet and not to have to go to a health centre or clinic.	83.0%	82.2%	76.7%
I can access help at any time that suits me.	94.0%	90.0%	87.2%
The computer will not criticize me.	63.2%	58.9%	65.6%

### Comparisons

**Figure 4:**
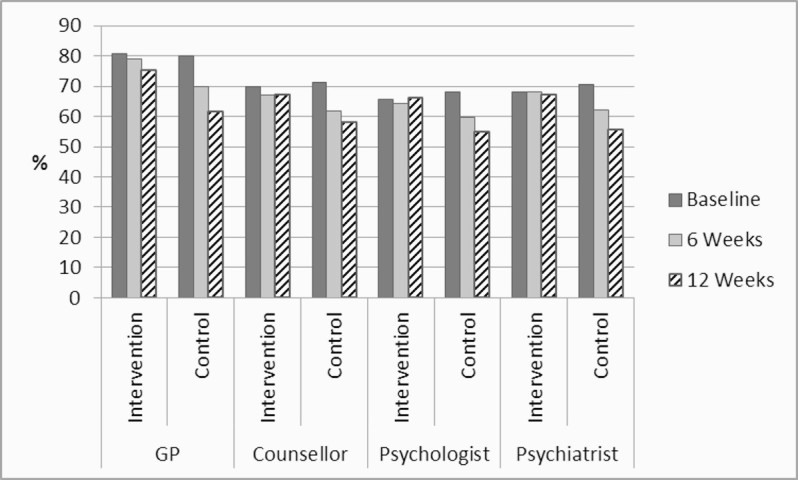
Comparisons between online self-help and face-to-face professional help.

Figure 3 shows how the comparisons with professional inputs were measured, and Figure 4 shows how they differed between control and intervention group. At the outset, a majority of respondents regarded online self-help to be equally or more acceptable than seeing health care professionals face to face. This held true at 6 weeks and 12 weeks across both study arms. However, unlike the intervention group, the control group’s ratings decreased significantly at 6 weeks in relation to three of the four alternatives: GP (*t*_189_=2.472, *P=*.014), counsellor (*t*_189_=2.206, *P=*.029), psychologist (*t*_189_=1.527, *P=*.129), and psychiatrist (*t*_189_=2.267, *P=*.025). The 12-week data showed that the difference between the ratings of intervention and control participants was sustained over time. Again, responses were highly intercorrelated at all points in time.

### Expectations and Experience

The open-ended comments in response to “Any other reasons to like or dislike help via the Internet: please give brief details” provide insight to interpret respondents’ views; 284 participants (78%) responded to the open question (142 of them from each arm). At 6 weeks, 98 participants (51 from the control condition) commented again (30% of those retained in the study), and 28 at 12 weeks (20 from the control condition), which was 12% of the number who responded at this time point. Edited slightly to protect anonymity, representative comments by study arm at baseline are given below, distinguishing between comments from people in the control (“C”) and experimental (“E”) arms of the trial.

**Table 3:** Representative comments from control and intervention group members.

Group	Comments
**Control group**	
	*Dislike psychoanalysis by computer because it is impersonal, and human conditions do not readily fit computer program algorithms so accurate or appropriate diagnosis is a hit and miss process.*
	*I can print things off and read them at my own pace—as well as ensuring that I understand what is going on. The only issue is still feeling very alone because the computer is not a person.*
	*I feel that if you think you have a slight problem, then this is a good first step. If you feel like there is more treatment needed then other avenues can be looked at. This is a non-committed [sic] way of dealing with a problem.*
	*In theory I like help via the Internet, but to be honest I have found this program completely useless. It was not what I expected at all. All it did was provide me access to websites which are the sort of thing I can find easily on the Internet anyway.*
**Intervention group**	
	*I have struggled to find the time / prioritize with a very high workload. I should have done more modules and prioritized this higher.*
	*Just the fact it’s there when you need it, although does not suggest to get help if trend is getting worse. The questions didn’t ask about any traumatic/stressful events during the week…*
	*The Internet is a useful way of accessing information and help, but there will always be occasions when it could not replace talking to a person face to face.*
	*There were a number of the personal logs that were unclear and needed to be discussed with someone before completion. This meant that I didn’t necessarily get the best out of the program.*

At baseline, 40 people (14%) felt that they could not yet comment or made statements that were neither positive nor negative, and dropout at 12 weeks was high (63%, 406/637); therefore, the 98 comments given at 6 weeks are the best indicator of experience of using the websites. Negative comments outnumbered positive across the study by about 2.5 to 1, with intervention and control participants generating a similar ratio of negative to positive statements, while the reasons given for dissatisfaction vary widely, some reflect the different arms of the trial: “This ‘course’ which I was on was no help to me at all. There was no interaction with the program and I feel let down by it” [C] and “I have struggled to find the time / prioritize with a very high workload. I should have done more modules and prioritized this higher”. [E]

Completion of online sessions for the control group was not monitored, but for the intervention group the mean number of sessions completed out of a possible 20 was 8.35 (SD 6.76). This did not differ significantly between the three workforces.

### Participants’ Views of the Process

Participants came from a wide range of occupational backgrounds and ranged from those who rejected the use of the term “mental health problem”, for example, “I consider myself to be stressed due to excessive work load—I am 100% sane and still in control—albeit struggle with corporate bullying at work. Mental health problem reminds me of Film One Flew Over the Cuckoo’s Nest” [E], to those who had extensive experience of mental health services: “Materials viewed over the past few weeks have all been fairly basic, and having coped with depression for the last 30 years some of it was not new info.” [C]

Altogether, 400 comments of all kinds were submitted at all three time points. These fell into four broad categories: (1) intrinsic, or individual barriers that people faced to participating fully in the trial, (2) extrinsic issues concerning the structure or content of the websites or the context of their use, (3) generic judgments or generalizations about online self-help with only indirect reference to the respondents’ experience in the trial, and (4) specific opinions about MoodGYM or the control condition.

### Intrinsic Barriers

Regardless of which study arm they followed, a number of respondents were negative towards computer use from the start: “I use the computer almost 100% of the time at work. Using it to reduce my stress and depression hasn’t been helpful, in fact I found it stressful.” [C]

By contrast, doing things online was seen as familiar, convenient, and “relaxing” by others: “[I]t gives you a sense that you’re not really having a real mental health issue when using your PC online at home, as its very relaxed etc, whereas visiting a health center would probably bring home the notion that you are actually having a real problem.” [E]

Other intrinsic barriers included lack of time and distractions. In addition, depression brings its own obstacles to self-help, for example, low self-esteem, apathy, and difficulty concentrating. Several people in both arms acknowledged that their motivation to complete the program was low: “Can be too tired to bother with and when feeling low what’s the point?” [C] and “It can be impersonal and it is too easy to ‘slack off’ some of the exercise”. [E]

There were indications that that the ease of access of online resources could help to overcome some of the inertia of depression: “My GP recommended seeking CBT support through the NHS, which just seems too difficult to coordinate and organize right now, while trying to stay functional at work. Online access to support means I can do something to take a step in the right direction…” [E]

Yet participation was seldom entirely easy and straightforward. The following comment reminds us that the control condition consisted of informational websites, in contrast to the intervention, which offered a more interactive package: “When you are pressured at work and it’s all getting too much, the last thing you have time for or want to do is go on the computer and search websites.” [C] At the same time, this response illustrates the interaction between workload, stress, and the inertia often associated with depression.

### Extrinsic Barriers

Practical obstacles to access are treated here as “extrinsic” barriers. Some dissatisfaction with the online self-help approach was due to avoidable technical issues, as a small proportion of people mentioned that they encountered recurrent difficulties, for example, the “page kept expiring”. Several people made suggestions about how the experimental interface could be improved to promote accessibility and their own engagement with the process. For example, “The interaction between myself and the online system is not great. The questions are not likely to change on the strength of the answers I give so a predetermined path will be followed regardless. There are occasions where assistance could be given.” [E]

The comment that “the questions are not likely to change” seems to be a criticism of the extent to which any program can be tailored to individuals, or is presented as being adaptable, thus personalization of the online materials could be an area for development.

An issue affecting the delivery of online interventions was raised by a couple of respondents who had dyslexia: one mentioned that screen reader software (a text-to-speech application) could have improved the trial’s accessibility for people with this kind of problem.

As a means of overcoming some intrinsic barriers, people suggested adaptations such as auto-links embedded in emails to prompt them to log on or to complete exercises, making it harder for people to “forget” to use the program. Most extrinsic barriers would be amenable to improvements.

### Generic Judgments

The largest single category of critical comments (93/404, 23% of all comments at baseline, 6 or 12 weeks) concerned the impersonal nature of the online approach, such as, “I dislike using the Internet intensely as most issues relating to anxiety, depression, and stress at work are relational in nature and therefore best dealt with in relation to at least one other person instead of a piece of machinery!!” [E] and “Computer is impersonal, asks a predefined set of questions that may not be relevant to me.” [C]

However, 50 people (12.5%) actively preferred an indirect approach, due to embarrassment, shyness, fear of being judged, or of repeating painful encounters: For example, “No eye contact, less chance of me crying” [E] and “Previously, talking about my situation person-to-person had made me very emotional. Answering questions on the Internet does not elicit the same response.” [C]

While recognizing the drawbacks, such participants felt that on balance, the computerized approach suited them in their particular circumstances. To this group may be added a smaller number (15, 4%) who stated that the anonymity of the approach was an advantage: “When you are in a high profile job, a computer doesn’t care who you are.” [C] and “My main fear if I were to seek help face to face would be the impact this would have on my self-esteem.” [C]

Positive opinions were also expressed about the ease of access, range of information, and structure offered by the online approaches by 32 people (8% of all comments at baseline, 6 or 12 weeks). In total, over all data collection points, there were 24% (97/404) of people who expressed positive opinions, compared to 23% (93/404) who found it unacceptably impersonal.

### Specific Responses

People in the intervention arm were more likely to express satisfaction with particular aspects, although with reservations in some cases: “I like the fact that I have a written record and can look back on the information. The tips and relaxing techniques give me a little bit of control in working towards a feeling of well-being.” [E]

A few specific issues were raised concerning the content of the intervention: “I did not find this tool helpful. The wording (perhaps translation) was poor, sounded pat…” [E] and “Not all results explained well. Some of written exercises quite laborious too.” [E]

Nonetheless, positive responses were more frequent: “It is a fantastic idea and the only thing that has really helped me” [E] and “I found the introduction of concepts I didn’t know about and they were useful apply to my own thinking.” [E]

As noted, some people in the control arm were clearly dissatisfied with the websites they were offered, for example “can’t find a user-friendly website” and “I have found this program completely useless”, although there were also more moderate views such as “a good first step” or “you can gather comparative information from different sources” and one citing the scope and flexibility of online resources. “Sadly, this course of ‘self-help’ did not give me any HELP, only access to information which I already knew how to access. I felt it was a waste of my time, although I kept it up in the hope that next week may show an improvement in satisfying my needs.” [C]

## Discussion

### Principal Results

The main finding of this analysis is that employees with diagnosable depression who chose to use online self-help were broadly positive about the experience when asked to rate it, although they identified a number of areas for improvement. Despite the fact that negative comments outnumbered positive by 2.5 to 1, more than 60% of people who were willing to try the online approach considered it to be at least as acceptable as seeing a professional about mental health issues. It is important to note that they were more likely to retain this opinion over time if they used the interactive program, rather than informational websites alone.

The respondents commented on barriers to engagement with the online resources, which included psychological and emotional impediments together with imperfections in the technical aspects of the websites and the barriers to their use faced by people with dyslexia. This provides background information about the perceived advantages and disadvantages of cCBT, over which our respondents were fairly equally divided. While some found the Internet non-threatening, convenient, and anonymous, a similar number felt that what they needed most was someone to talk to. These results echo previous studies, including a systematic review of the drivers of adherence to Internet trials [19-21]. In particular, the findings show that many participants were facing logistical, psychological, and emotional barriers to seeking help in person from conventional professional sources. This indicates a reservoir of need that cCBT could potentially meet more effectively than standard treatment approaches.

From a methodological perspective, the views of such a substantial number of users are a valuable resource in developing online interventions to make them more user-friendly, accessible, acceptable, and hopefully more effective. The findings also have relevance for researchers seeking to gather data online, indicating, for example that while some topics are more amenable to face-to-face interviewing, there are people who are willing to engage with online approaches.

### Limitations

Participants were self-selected; people who find online self-help unacceptable would never have considered taking part in this trial. The dropout rate from this study (45% at 6 weeks) was high, meaning that the inferences drawn here about the acceptability or effectiveness of online interventions come mainly from people who were motivated to complete the course. It might have been preferable to include interactive materials as the attentional control, making this more similar to MoodGYM. Some dissatisfaction reported here clearly relates to the discontinuity between the informational websites provided to the control group. While it is regrettable that the content of the control condition caused frustration, this should not reflect badly on the experimental intervention. The materials offered in each arm inevitably suited some people better than others, and the potential range of resources that people can access online is bound to be larger than that available to clients of a single counselor, especially those on time-limited courses of treatment. This suggests that expert appraisal and selection of resources for a range of purposes would be helpful. From a methodological perspective, these findings underline the importance of having a carefully designed active control group in RCTs of this kind.

### Implications

It is possible that dropout indicates dissatisfaction. Retaining people in online interventions for longer is one means to increase their exposure to the therapeutic effects, and therefore to the potential benefits [20]. Yet, since costs of delivering online programs are generally low by comparison with face-to-face counseling, the diversion of any consumers from the latter to the former represents a potential saving. A middle path appears to be online therapy supported by some professional input. We used clinical studies officers simply to monitor and troubleshoot adherence to the intervention, while Warmerdam et al [22] utilized a life coach with a similar function, and Mohr et al in a feasibility study, provided manualized telephone and email support provided by “PhD-level licensed psychologists” [23].

The comments presented here confirm that online self-help does not suit everyone, but as one participant said “it certainly has its place”. At the point of delivery, careful management of users’ expectations is needed to ensure that online self-help is not seen disparagingly as a less costly and inferior alternative to interpersonal therapy. Online interventions are continually evolving; adapting them to meet individual need seems to be the key to success. More knowledge is needed about the characteristics of people who are best suited to online self-help in order to predict who is likely to benefit [24].

We suggest that the acceptability of online self-help could be improved in three ways. First, at the individual level, effective engagement could be promoted by investigating the intended consumers’ expectations and concerns and then exploring how their particular needs can be met. For instance, offering back-up advice by telephone might be one way to encourage those who are reluctant users. This could be delivered by different levels of staff according to the users’ needs. Networks of peers who have used the same online approaches could be developed to offer the longer-term support that some people seek to respond to the evident need for more human contact. Second, it is reasonable to expect that adaptations for sensory impairment and dyslexia should be incorporated into existing and new online packages. Finally, at the systemic level, expectations of cCBT could be actively shaped by public health initiatives and social marketing with reference to the wider evidence base.

There is further research to be done to ascertain whether approaches combining face-to-face with online interventions can satisfy the need expressed for person-to-person encounters to overcome their mental health problems—and if so, how much personal contact is enough in combination with online CBT.

### Conclusions

The following comments sum up the views of the respondents: “It’s a little TOO impersonal but it’s an excellent complementary way to handle emotional/psychological issues and to provide self-help techniques. I don’t think this can replace the more usual means of obtaining help but it certainly has its place” [C] and “Help via the Internet is far more accessible than having to wait on a GP referral to a counselor. Some emotional problems can certainly be helped by following a CBT program on the Internet.” [E]

Our conclusion is that online, self-help resources for depression will help some people but not all. Clearly they cannot replace face-to-face therapy but may be a useful adjunct in certain circumstances. Many providers already include structured self-help as an integral part of treatment pathways of care for common mental disorders, and the findings of this study support that strategy. Barriers to use of online self-help resources may relate to individual perceptions and expectations, public opinion, or to the nature of the online resources. Attention to the detailed findings presented here could help reduce the typically high dropout rates from online interventions in general.

To the extent that people who might otherwise have been on waiting lists for face-to-face services found the use of online resources helpful, they offer potential benefits. Not everyone believes they need face-to-face counseling. An important conclusion from this study is that some people in employment with depression simply prefer a “faceless” approach. With the caveat that screening for immediate risk should be provided, the broad acceptability of online interventions makes them a promising option for many working adults who experience depression.

## Multimedia Appendix 1

Consort diagram.

## Abbreviations

CBT: cognitive behavior therapy
cCBT: computerized cognitive behavior therapy
CSO: Clinical Studies Officer
GP: general practitioner
NHS: National Health Service
PHQ: Patient Health Questionnaire
RCT: randomized controlled trial
WSAS: Work and Social Adjustment Scale
