# Supplementary figures and images for: Acceptability of Online Self-Help to People With Depression: Users’ Views of MoodGYM Versus Informational Websites
Source: J Med Internet Res. 2014 Mar 28;16(3):e90. doi: 10.2196/jmir.2871 (PMC4004160; doi:10.2196/jmir.2871)

Multimedia Appendix 1. Consort Diagram.

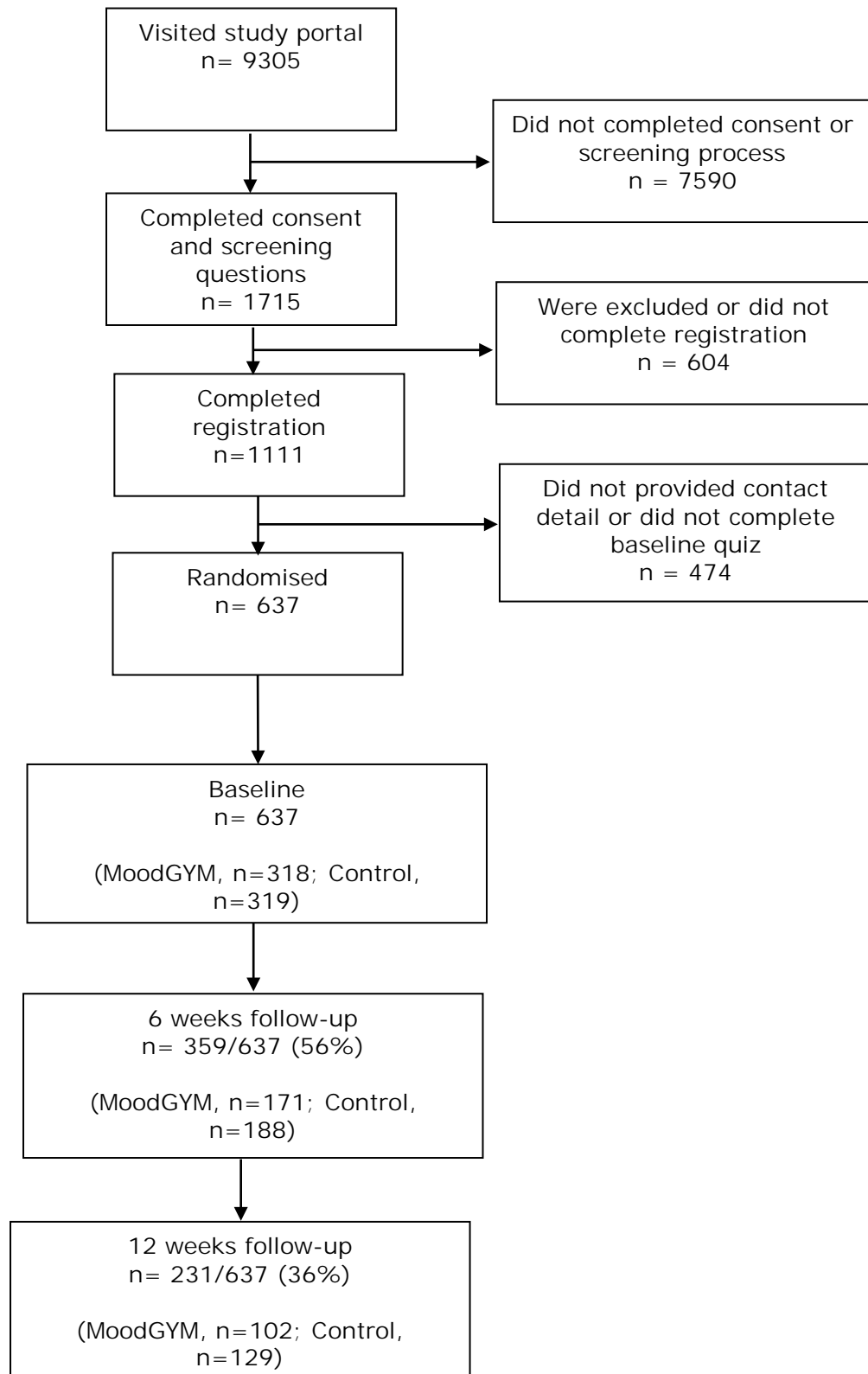

Supplement: Supplementary file 1 [file jmir_v16i3e90_app1.pdf]
